# Effectiveness of virtual mindfulness-based interventions on perceived anxiety and depression of physicians during the COVID-19 pandemic: A pre-post experimental study

**DOI:** 10.3389/fpsyt.2022.1089147

**Published:** 2023-01-09

**Authors:** Abdullah Al Ozairi, Dalal Alsaeed, Ebaa Al-Ozairi, Mohammad Irshad, Rebecca S. Crane, Aroub Almoula

**Affiliations:** ^1^Faculty of Medicine, Kuwait University, Kuwait City, Kuwait; ^2^Amiri Hospital, Ministry of Health, Kuwait City, Kuwait; ^3^Dasman Diabetes Institute, Kuwait City, Kuwait; ^4^Centre for Mindfulness Research and Practice, Bangor University, Bangor, United Kingdom

**Keywords:** anxiety, depression, physicians, healthcare profession, mindfulness-based interventions (MBIs)

## Abstract

**Background:**

The outbreak of coronavirus disease 2019 (COVID-19) created unprecedented stress on physicians. Mindfulness is a type of meditation that focuses on being fully present, aware of senses, and emotions in the present moment without analyzing or judging them, and it may help reduce psychological distress in physicians. This study aimed to examine the effectiveness of virtual mindfulness-based intervention (MBI) on physicians’ perceived anxiety and depression and different facets of mindfulness.

**Methods:**

During the COVID-19 pandemic, an online survey was administered to physicians to assess depression, anxiety, and awareness using the 9-item Patient Health Questionnaire (PHQ-9), 7-item General Anxiety Disorder (GAD-7), and Five-Facets Mindfulness Questionnaire (FFMQ), respectively. Physicians that received the virtual MBI sessions also completed post-questionnaires at a 3-week follow-up time point.

**Results:**

A total of 125 physicians responded to the online survey, with 56 completing the MBI. The prevalence of moderate to severe anxiety and depression was 45.0 and 46.7%, respectively. Mindfulness scores were negatively associated with depression (*r* = −0.38, *P* < 0.001) and anxiety (*r* = −0.36, *p* < 0.001). Mindfulness scores for the 56 physicians who received virtual MBI sessions were significantly improved (mean difference ± SD, 17.7 ± 16.1, *p* = 0.001). Significant reductions were also evidenced in anxiety (4.4 ± 4.2) and depression (4.5 ± 5.1) scores (*p*’s < 0.001). There was also an improvement in mindfulness facets of observing (5.1 ± 4.7), describing (2.3 ± 4.3), acting with awareness (2.7 ± 5.3), non-judging of inner experience (3.6 ± 6.1), and non-reactivity to inner experience (3.9 ± 4.0) (*p*’s < 0.001). A facet of mindfulness, acting with awareness was most efficiently associated with improved anxiety (*B* = −0.3, *p* = 0.02) and depression (*B* = −0.4, *p* = 0.01).

**Conclusion:**

This study has demonstrated that virtual MBI improved physicians’ psychological wellbeing and mindfulness during the crisis. Regular mindfulness practice may help physicians to tolerate and handle unpleasant circumstances, such as future epidemics or pandemics.

## 1. Introduction

The outbreak of coronavirus disease 2019 (COVID-19) in December 2019 in Wuhan City, China (1), quickly created a catastrophic situation worldwide (2). The United Nations considers this crisis one of the most traumatic or challenging events for human beings since World War II. COVID-19 has increased the demand and pressure on the current healthcare resources globally, leading to healthcare resource shortages. Frontline physicians and other healthcare workers have reported being worried about contracting COVID-19, carrying the virus at home, and infecting their loved ones, including aging parents, infants, and relatives (3). They experienced feelings of extreme vulnerability, uncertainty, life threat, and somatic and cognitive anxiety symptoms (4). Consequently, healthcare professionals psychological stress has increased enormously, especially in those directly dealing with patients suffering from COVID-19 (5). Poor mental and physical health among physicians and other healthcare workers during the COVID-19 pandemic has been widely reported to negatively affect their emotional and social wellbeing (6, 7). Levels of psychological distress, depression, anxiety, and sleeping difficulties during the COVID-19 pandemic period were significantly elevated (8–10) and it is known that severe levels of psychological stress can indeed impair the performance of healthcare workers and adversely affect their attitudes and behaviors (11, 12).

Pandemic-like circumstances may, therefore, warrant psychological assistance for frontline physicians and other healthcare workers to alleviate anxiety, stress, and sleep difficulties. Results of several reviews highlight that several workplace-based interventions have been implemented to support healthcare workers’ psychological and emotional wellbeing (13–15). Such programs commonly provide essential need resources or services, workplace training programs, peer-support programs, and psychoeducational or counseling services to mitigate healthcare workers’ psychological situations. However, researchers have not previously considered methods that may ensure the long-term benefits of programs (13, 14), such as mindfulness techniques.

During the COVID-19 pandemic, many forms of mindfulness-based practices have gained popularity to reduce psychological stress and anxiety and enhance the sleep quality of healthcare workers (16, 17). For example, the online Mindfulness Ambassador Program has increased the resiliency of healthcare workers (16) and mindfulness-based breathing and music therapy have mitigated stress and work-related strain (17). Many mindfulness-based interventions (MBIs) can enhance emotional regulation and reduces symptoms of various mental health disorders (18–20).

Mindfulness meditation practice originated from the ancient Buddhist tradition and philosophy of paying attention to purpose in the present moment without judgment (21). Later, mindfulness principles were secularized by recontextualizing them from their original cultural, religious, and ideological elements associated with Buddhism. The first modified form of mindfulness was developed for the western mind and culture, known as mindfulness-based stress reduction (22). Nowadays, various forms of mindfulness meditation have been developed, but unfortunately the structure of most mindfulness programs is either not well planned or inappropriate for the Arabic culture. Therefore, we sought to adapt MBI appropriately to the Arabic culture.

The present study used a modified, structured mindfulness meditation program adapted from mindfulness-based cognitive therapy (MBCT). This program may help to reduce various mental health problems and improve the facets of mindfulness. These facets include “observing” (noticing or attending to internal feelings and thoughts and external simulation), “describing” (labeling feelings, thoughts, and experiences with words), “acting with awareness” (attending to what is currently happening), “non-judging of inner experience” (taking a non-evaluative stance toward internal thoughts and feelings), and “non-reactivity to inner experience” (allowing emotions and thoughts to come and go, without being interfered by them) (23, 24).

This study is the first to execute mindfulness sessions in Arabic physicians and examine MBI’s impact on the five facets of mindfulness and the level of recovery from depression and anxiety during the COVID-19 pandemic. We hypothesized that the mindfulness sessions would help elucidate the relationship between the changes in the five facets of mindfulness and physicians’ psychological symptoms outcomes, such as anxiety and depression. Results of the current study may encourage Arabic people to accept mindfulness meditation.

## 2. Article types

A pre-post experimental study

## 3. Materials and methods

### 3.1. Study procedure

This is a quasi-experimental study conducted during the COVID-19 pandemic (2020–2021). Physicians (*n* = 126) were invited to participate in MBI and then asked to complete an online survey to measure anxiety, depression and mindfulness pre-intervention and a follow-up survey 3 weeks after the completion of MBI.

Virtual MBI sessions were provided virtually in a group *via* zoom. We assigned participants to different online virtual groups based on availability and job schedule. The inclusion criteria were healthcare physicians employed by the Kuwait Ministry of Health or affiliated institutions. Those who did not speak Arabic were excluded from the MBI participation. The participation was entirely voluntary.

An anonymous, self-administered, and bilingual questionnaire (Arabic and English) was employed to measure anxiety, depression, and mindfulness levels. The data were collected at two time points before and after 3 weeks after MBI using a web-based online survey. This study was approved by the Institutional Review Board of the Kuwaiti Ministry of Health, which conformed to the principles of the Declaration of Helsinki. The physicians provided written informed consent.

### 3.2. Mindfulness-based intervention (MBI)

Using an online videoconferencing platform, a clinical psychologist and a psychiatrist virtually provided MBI to physicians in Arabic. MBI took place into many groups and each group of participants received MBI sessions 2 h a day, for eight sessions during 2 weeks period. These sessions were facilitated by an experienced psychiatrist (AO) and a clinical psychologist (AA) trained in mindfulness from Bangor University’s Mindfulness Centre, UK. At the end of the sessions, the director (RSC) of Bangor University’s Mindfulness Centre offered a post-workshop session to all participants. All these sessions were live, and recordings were shared with the participants.

Participants received an explanatory introduction to MBI. Each intervention session emphasized (1) focused attention by paying attention to a specific anchor, such as mindful breathing, sitting meditations, body scans, or sound meditations, both internal and external; (2) conscious movements through walking meditation and mindful walking; and (3) informal practices to aid compassion and kindness by using an inviting attitude and language (i.e., using specific sentences and gestures that invite care for oneself and others).

In addition, mindfulness audio recordings (e.g., sitting meditation) were provided to participants as a written script and audio recording, as well as a brief description of mindfulness facets to read and practice at home. In order to encourage participants to complete the program on a daily basis, the researchers also provided them with a log sheet each week to record practices and make a note of anything that came up when they practiced. An encrypted HIPAA-compliant group chat was also created for each group to discuss daily activities and duties, answer any questions participants may have, and provide opportunities for exchanging experiences.

The PI monitored the intervention and ensured that each protocol component was delivered. All sessions of MBI were video recorded to ensure the fidelity of the intervention (Supplementary Annexure 1). In addition, a checklist of homework and pleasant and unpleasant events were discussed in the proceeding sessions, as is the norm in MBI protocol.

### 3.3. Measures

The measures were administered in the following order: General Anxiety Disorder-7 (GAD-7), Personal Health Questionnaire-9 (PHQ-9), and Five-Facets Mindfulness Questionnaire (FFMQ).

### 3.4. Assessment of mindfulness

The FFMQ was used for assessing mindfulness. FFMQ consists of 39 items with responses based on a 5-point Likert scale, between 1 (never or rarely true) and 5 (very often or always true), and the total score ranges from 39 to 195 (25). Higher values indicate additional mindfulness skills. The FFMQ measures the five subscales of mindfulness: observing, describing, acting with awareness, non-judging of inner experience, and non-reactivity to inner experience. The internal reliability (α) of the FFMQ questionnaire before intervention was 0.89 (for the five subscales, 0.69–0.88), and after intervention was 0.91 (for the five subscales, 0.71–0.90), suggesting an acceptable level of internal reliability.

### 3.5. Assessment of depression

The PHQ-9 was used for assessing depression symptoms. The PHQ-9 consists of nine questions designed to measure the presence and severity of depressive symptoms as per the Diagnostic and Statistical Manual of Mental Disorders-IV criteria (DSM-IV) (26). Each question is scored between 0 (not at all) and 3 (nearly every day), and the final score ranges from 0 to 27. The final score of PHQ-9 is 10 or more for positive depression symptoms and 15 or more for moderately severe depression symptoms (27). The internal reliability of PHQ-9 before intervention (α = 0.82) and after intervention (α = 0.83) were optimal.

### 3.6. Assessment of anxiety

The GAD-7 questionnaire was used for assessing anxiety symptoms. The GAD-7, which has seven questions, is widely used to assess anxiety levels (28). Each question scored between 0 (not at all) and 3 (nearly every day), and the total score ranges from 0 to 21; higher scores indicate greater severity. A GAD-7 score of 10 or more indicates positive anxiety symptoms, and 15 or more indicates severe anxiety symptoms. The internal reliability of GAD-7 before intervention (α = 0.91) and after intervention (α = 0.88) were optimal.

### 3.7. Statistical analysis

Data were analyzed using SPSS (version 25.0, IBM SPSS Statistics; IBM Corp., Armonk, NY, USA). Kolmogorov–Smirnov tests were applied to assess normality. Descriptive statistics, mean (standard deviation), or proportions (%), for the whole sample and those who participated in MBI were calculated. Pearson correlation analyses were used to explore the association between the five mindfulness facets and anxiety or depression. Paired *t*-test was used to compare the pre- and post-test measures of mindfulness, anxiety, and depression. Mann–Whitney *U* test was used to compare between the sex. The Chi-square test was used to examine the differences between categorical variables. Regression analyses were used to identify facets of mindfulness that predicted reductions in anxiety and depressive symptoms following MBI intervention. The effect sizes were calculated through Cohen’s d, and values of 0.20, 0.40, and 0.60 indicate small, medium, and large effect sizes, respectively (29). Multicollinearity among the predictor variables was assessed using tolerance and variance inflation factor (VIF) statistics. A *P*-value of less than 0.05 was considered statistically significant.

## 4. Results

### 4.1. Baseline characteristics

**FIGURE 1:**
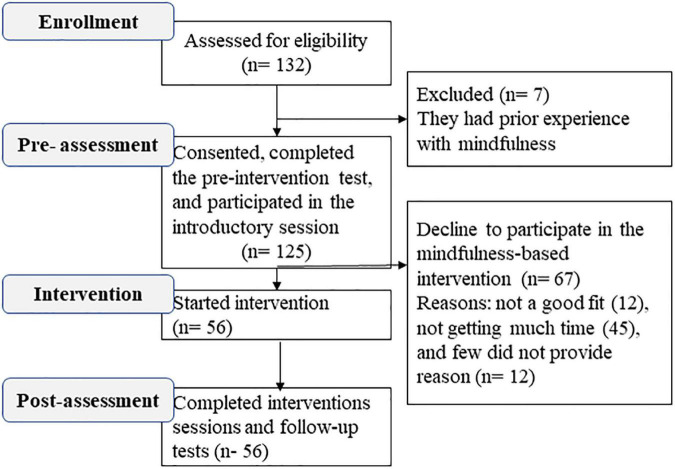
CONSORT flow diagram for participants in mindfulness-based intervention (MBI).

Figure 1 shows the flow of participants. A total of 125 physicians completed the online survey questionnaire. Of these participants, 79.2% were female, 45.0% had anxiety, and 46.7% had depression. The prevalence of moderately severe levels of depression was 21.3% and severe level anxiety was 18.0%. Fifty-six physicians participated in the virtual MBI sessions, 82.1% were female, 46.4% had anxiety, and 48.2% had depression symptoms. Table 1 reports the demographic characteristics and baseline symptoms.

**TABLE 1:** Demographic information of physicians.

Variables		Total respond (*N* = 125)	Participated in MBI (*N* = 56)
		*n* (%) or mean (SD)	*n* (%) or mean (SD)
Gender	Male	26 (20.8)	10 (17.9)
	Female	99 (79.2)	46 (82.1)
Education level	Graduate	44 (35.2)	41 (73.2)
	Postgraduate	81 (64.8)	15 (26.8)
Age (Years)	18–34	65 (52.0)	23 (41.1)
	35–44	42 (33.6)	22 (39.3)
	45–64	18 (14.4)	11 (19.6)
Depression	Moderately severe	31 (25.4)	15 (26.8)
	Severe	26 (21.3)	12 (21.4)
Anxiety	Moderately severe	33 (27.0)	16 (28.6)
	Severe	22 (18.0)	10 (17.9)
Mindfulness facets	Observing	24.5 (5.5)	25.1 (5.3)
	Describing	25.1 (5.6)	25.6 (5.6)
	Awareness	23.3 (6.0)	24.5 (5.2)
	Non-judging	22.8 (6.0)	21.8 (5.1)
	Non-reactive	18.0 (4.1)	18.9 (3.7)
	Total score	114.7 (16.2)	116.0 (13.5)

MBI, mindfulness-based interventions.

### 4.2. Correlation between depression, anxiety, and mindfulness in cohort sample

The mean score on the FFMQ was 114.7 (SD = 16.2), which was negatively correlated with PHQ-9 scores (*r* = −0.38, *p* < 0.001) and GAD-7 scores (*r* = −0.36, *p* < 0.001). The GAD-7 scores negatively correlated with the FFMQ facets scores of “describing” (*r* = −0.23, *p* = 0.011), acting with awareness (*r* = −0.33, *p* = 0.001), non-judging of inner experience (*r* = −0.35, *p* = 0.0001), and non-reactivity to inner experience (*r* = −2.0, *p* = 0.027). However, the PHQ-9 scores negatively correlated with the subscale scores of describing (*r* = −0.29, *p* = 0.001), acting with awareness (*r* = −0.30, *p* = 0.001), and non-judging of inner experience (*r* = −0.39, *p* = 0.0001). However, PHQ-9 score was not significantly correlated to mindfulness facets of observing and non-reactivity to inner experience.

### 4.3. The effects of MBI on facets of mindfulness, anxiety, and depression

**FIGURE 2:**
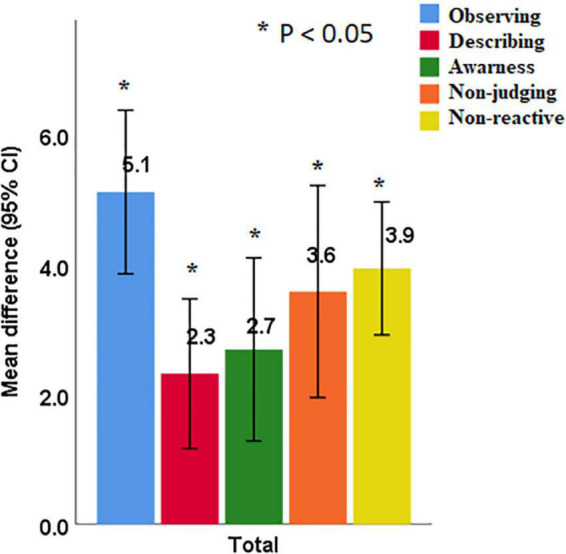
Improvement of mindfulness facets score after interventions.

Figure 2 represents the effects of MBI on five facets of mindfulness. The overall FFMQ scores significantly increased from pre- to post-intervention (116.0 ± 13.5 vs. 133.6 ± 16.8, *p* < 0.001) (Table 2). The scores of FFMQ facets of observing (mean differences ± SD, 5.1 ± 4.7), describing (2.3 ± 4.3), acting with awareness (2.7 ± 5.3), non-judging of inner experience (3.6 ± 6.1), and non-reactivity to inner experience (3.9 ± 4.0) significantly increased from the baseline to post-intervention (*p*’s < 0.001). FFMQ score significantly improved in women (18.5 ± 16.9, *p* = 0.001) and men (13.5 ± 11.3, *p* = 0.001). The effect size from pre- to post-test indicated a large intervention effect on mindfulness (*d* = 1.14). The effect size within the sex group also indicated a large intervention effect on mindfulness (*d* > 0.98).

**TABLE 2:** Mean difference of mindfulness, anxiety, and depression scores after the mindfulness-based intervention (MBI).

	Total (*n* = 56)		Women (*n* = 46)		Men (*n* = 10)		
	MD (± SD)	*d* ^†^	MD (± SD)	*d* ^†^	MD (± SD)	*d* ^†^	*P*-value^‡^
Mindfulness (FFMQ)	17.7 (16.1)**	1.14	18.5 (16.9)**	1.18	13.8 (11.3)**	0.98	0.50
Anxiety (GAD-7)	−4.4 (4.2)**	0.91	−4.7 (4.5)**	0.98	−3.0 (2.0)**	0.67	0.25
Depression (PHQ-9)	−4.5 (5.1)**	0.92	−4.9 (5.4)**	0.96	−2.5 (3.4)*	0.91	0.20

*t*-test- pre vs. posttest, *p < 0.01, **p < 0.001.

^†^Effect size pre-posttest.

^‡^Mann–Whitney *U* test between the sex.

After the MBI, anxiety (4.8 ± 4.2) and depression (4.5 ± 5.2) scores significantly decreased (*p*’s < 0.001) (Table 2). The decrease in anxiety and depression scores after the intervention was also significant in women and men (*p*’s < 0.01). The decrease in anxiety and depression scores between sex was not significantly different (Table 2). The effect size from pre- to post-test indicated a large intervention effect on the reduction of anxiety (*d* = 0.9) and depression (*d* = 0.92) scores. Moreover, within the sex group, a large effect was observed in the reduction of depression score, whereas a medium to large effect was observed in the reduction of anxiety score (Table 2).

In unadjusted linear regression analyses, describing, acting with awareness, and non-judging of inner experience were associated with positive change in depression, whereas acting with awareness was also associated with a positive change in anxiety (Model 1). However, after mutual adjustment of all five facets of mindfulness, a facet “acting with awareness” was significantly associated with positive changes in both depression and anxiety (Model 2). The collinearity test indicated a mild correlation between mindfulness facets (Table 3).

**TABLE 3:** Association of mindfulness facets with depression and anxiety.

Depression (PHQ-9)	Model 1	Model 2		
Mindfulness facets	*B* (95% CI)	*B* (95% CI)	Tolerance^†^	VIF^†^
Observing	−0.1 (−0.4, 0.2)	0.1 (−0.2, 0.4)	0.7	1.4
Describing	−0.4 (−0.7, 0.1)*	−0.2 (−0.5, 0.2)	0.7	1.4
Awareness	−0.5 (−0.7, −0.2)**	−0.4 (−0.7, −0.1)*	0.6	1.6
Non-judging	−0.3 (−0.5, −0.1)*	−0.1 (−0.3, 0.2)	0.7	1.5
Non-reactive	−0.1 (−0.5, 0.2)	0.0 (−0.4, 0.3)	0.8	1.2
**Anxiety (GAD 7)**				
Observing	−0.1 (−0.3, 0.1)	0.1 (−0.2, 0.3)	0.7	1.4
Describing	−0.2 (−0.4, 0.1)	0.0 (−0.3, 0.3)	0.7	1.4
Awareness	−0.3 (−0.5, −0.1)*	−0.3 (−0.6, −0.02)*	0.6	1.6
Non-judging	−0.1 (−0.3, 0.1)	0.0 (−0.2, 0.2)	0.7	1.5
Non-reactive	−0.2 (−0.5, 0.1)	−0.2 (−0.5, 0.1)	0.8	1.2

Model 1 is unadjusted. Model 2 is mutually adjusted. *p < 0.2, **p < 0.001.

^†^Collinearity test-tolerance and VIF, variation inflammation factors.

## 5. Discussion

Results of the current study evidence a high prevalence of anxiety and depression symptoms among physicians in Kuwait during the COVID-19 epidemic. The results found a negative relationship between anxiety and depression with mindfulness scores. The high prevalence of anxiety and depression symptoms among physicians during the COVID-19 crisis was consistent with many international (30, 31) and local studies (32, 33). While anxiety and depressive symptoms may result from several psychological factors, many studies have evidence that the central/primary reason for symptoms in the context of COVID-19 was fear of infection (30, 31). Moreover, fear of COVID-19 infection served as a mediator between the perceived risk and anxiety and depression (34). Overall, the high prevalence of stress and anxiety symptoms among physicians warrants additional support, including counseling and a break from work. Without counseling and support, healthcare workers might suffer from poor mental health, resulting in poor healthcare quality (35). MBI is a well-established practice for reducing anxiety, depression, and burnout and improving quality of life (19, 20).

Consistent with this, the current study demonstrated reductions in anxiety and depressive symptoms compared during the pre- and post-MBI intervention time points. The virtual MBI substantially enhanced the physicians’ five facets of mindfulness. These results are in line with the most recent evidence, which indicates that mindfulness-based training can significantly increase scores on self-reported mindfulness measures (36). A study suggested that mindfulness practice could improve brain function in response to an interoceptive challenge and may support optimal performance (37). Another possible explanation is that people who practice mindfulness can better focus on the present situations non-judgmentally, break connectivity to their dysfunctional cognitive patterns, and disengage from their current perceptions, emotions, and distressing behaviors.

Virtual MBI possibly enhances mental health skills and awareness in the present moment, as reported previously by researchers in other healthcare workers (20, 38). Practicing mindfulness helps individuals tolerate and handle negative experiences and assess them with an open mind and compassionate attitude. In addition, acceptance processes stimulated by mindfulness practice could also reduce negative effects (39, 40). The mindfulness scores significantly improved in both sexes. However, MBI has a slightly more positive impact on women than men. The reason may be that the women were more willing to participate in the MBI sessions than the men. A study also reported that women had more mindfulness and self-compassion than men due to more favorable response to meditation training (41).

This study showed that the improvement in mindfulness was associated with a positive change in physicians’ anxiety and depression symptoms. This result is in line with a recent comprehensive review, which revealed that mindfulness after MBI practice could mediate or be associated with improvements in several psychological health outcomes in people with anxiety and depression symptoms (24, 42). A recent study reported a positive impact of MBI in decreasing stress levels and burnout in healthcare workers during the COVID-19 pandemic (43, 44). The improvements in the five facets of mindfulness and psychotic symptoms following MBI could be a therapeutic effect of the intervention on reducing psychological symptoms through mindfulness enhancement (24). The benefits of MBI are assumed to emerge from the improvement of awareness at the present moment, decrease in automatic thought patterns, and breaking of the link between an unpleasant occurrence and ongoing thinking of the pandemic. MBI teaches mindfulness skills to increase intentional attention, develop a different relationship with one’s thoughts, and practice different strategies in relation to distressing thoughts and emotions non-judgmentally (45). In addition, mindfulness sessions include sitting meditation, mindful walking, and informal exercises. Such activity likely influences many biological and psychological mechanisms that may reduce or prevent psychological symptoms (46). The analysis of effect size on post-test outcomes within the sex group indicated a medium to large effect of the MBI on anxiety, whereas a large effect on depression. A study found a medium to large favorable effect of MBI compared with control groups on mindfulness, anxiety, and depression in traumatized young adults (47).

Regarding the association between mindfulness facets and depression, describing, acting with awareness, and non-judging inner experiences were protective factors of depression symptoms. However, acting with awareness was the most protective factor against anxiety and depression symptoms. Previous research reported that all mindfulness facets except observing were associated with improving depressive symptoms (48–50). The mechanisms by which mindfulness facets impacted psychopathological outcomes are unclear. Few researchers assumed that mindfulness breaks the ruminative cycle and makes them aware of their feelings and thoughts without judging them or getting trapped in them (44, 45), which helps to reduce depression symptoms. In addition, no significant effect of sex was found on improving anxiety and depression symptoms. The men sample size was remarkably less than women, so the effect of MBI within the sex group should be interpreted with caution.

This study has several limitations worth acknowledging; it was a single-arm, study of only 56 participants, and it cannot address the causality of the emotions. In addition, the high refusal rate for participation in MBI also indicates the low acceptance of MBI in the current setting. The measurement of mindfulness, anxiety and depression were exclusively online self-report measures, which can lead to reporting bias. Another limitation of this study was that the follow-up data after the 3 weeks of intervention was not collected. Thus, we cannot answer the lasting effect of MBI in the current situation. A large sample-based randomized trial study is required to explore the clinical applications of MBI. In addition, long-term research is needed to examine whether the effects of virtual MBI on mindfulness, depression, and anxiety are maintained over time or not.

Despite these limitations, this study yields promising results of MBI during the COVID-19 period. MBI improved mindfulness and reduced anxiety and depression symptoms. The results suggest that practicing mindfulness could reduce the symptoms of mental health conditions and the comorbid risk of physical health complications. These benefits of mindfulness may be helped to improve the self-compassion practices of healthcare professionals, especially in pandemic-like situations. In addition, the promising results may build interest for mindfulness practice in Arab culture.

## 6. Conclusion

Mindfulness-based interventions improved the physicians’ anxiety and depression, possibly related to the increase in the levels of different aspects of mindfulness. Practicing mindfulness could help physicians to tolerate and handle unpleasant circumstances, such as future epidemics or pandemics. Thus, this study recommends the practice of mindfulness in healthcare and training environments.

## Data availability statement

The raw data supporting the conclusions of this article will be made available by the authors, without undue reservation.

## Ethics statement

The studies involving human participants were reviewed and approved by the Institutional Review Board of the Kuwait Ministry of Health, Kuwait. The patients/participants provided their written informed consent to participate in this study.

## Author contributions

AAO developed the study design and concept, conducted the training, collected the data, and drafted the manuscript. DA and AA contributed to the participant recruitment, conducted the training, and acquired the data. MI handled the data analysis and drafted the manuscript. EA-O handled data analysis, interpretation, and wrote the manuscript. RC handled the post-workshop mindfulness training and critically review the final draft. All authors have read and given their approval to the final manuscript.
