# Supplementary material for: Effectiveness of virtual mindfulness-based interventions on perceived anxiety and depression of physicians during the COVID-19 pandemic: A pre-post experimental study
Source: Front Psychiatry. 2023 Jan 9;13:1089147. doi: 10.3389/fpsyt.2022.1089147 (PMC9868838; doi:10.3389/fpsyt.2022.1089147)
Supplement: Supplementary file 1 [file Data_Sheet_1.docx]

**Annexure 1**

We implemented several strategies to maximize intervention fidelity. The structure of an intervention comprises five elements essential for the fidelity of this study.

1. Design: MBI took place in many groups, and each group of participants received MBI sessions two hours a day, a total of eight sessions during the two weeks.
2. Training: All sessions were delivered by trained instructors who received training from Bangor University's Mindfulness Center, UK, and backup by a clinical psychologist (PI). The other members (facilitators) received the scripted presentation from the intervention manual.
3. Delivery: The PI monitored the delivery of interventions (8 sessions) and reviewed the recorded video to ensure the instructors' adherence to the protocol. A checklist of homework and pleasant and unpleasant events was discussed in each proceeding sessions.
4. Receipt: Participants received reminder messages one day before and just before the sessions. The attendance of the participants was recorded in each session. MBI was offered during the COVID-19 lockdown, so all participants attended all sessions (100% attendance). PI and instructor were readily available by phone and email to answer the participant's query.
5. Enactment: The performance of MBI practice was assessed by using the five facets of the mindfulness questionnaire (FFMQ), anxiety (GAD-7), and depression (PHQ-9) scales.

**Pleasant Events Calendar (**Become aware of your thoughts, feeling, and body sensations around one pleasant event each day)

| What was the experience? | Were you aware of the present feelings while the event was happening? | How did your body feel, in detail, during this experience? | What moods, feelings, and thoughts accompanied this event? | What thoughts are in your mind now as you write about this event |
| --- | --- | --- | --- | --- |
| Sunday |  |  |  |  |
| Monday |  |  |  |  |
| Tuesday |  |  |  |  |
| Wednesday |  |  |  |  |
| Sunday |  |  |  |  |
| Monday |  |  |  |  |
| Tuesday |  |  |  |  |
| Wednesday |  |  |  |  |

**Unpleasant Events Calendar**

Be aware of an unpleasant event at the time it is happening. Use these questions to focus your aware on the details of the experience as it is happening.

| What was the experience? | Were you aware of the unpleasant feeling while the event was happening? ? | How did your body feel, in detail, during this experience? | What moods, feelings, and thoughts accompanied this event? | What thoughts are in your mind now as you write this down? |
| --- | --- | --- | --- | --- |
| *Example: Waiting for the cable company to come out and fix our line. Realize that I am missing an important meeting at work.* | *Yes.* | *Temples throbbing, tightness in my neck and shoulders, pacing back and forth*. | *Angry, helpless. “is this what they mean by service? they don’t have to be responsible, they have a monopoly.” this is one meeting / didn’t want to miss.* | *I hope I don’t have to go through that again soon*. |
|  |  |  |  |  |
|  |  |  |  |  |
|  |  |  |  |  |
